# Self-reported psychopathic traits among non-referred Finnish adolescents: psychometric properties of the Youth Psychopathic traits Inventory and the Antisocial Process Screening Device

**DOI:** 10.1186/s13034-015-0047-6

**Published:** 2015-06-06

**Authors:** Svetlana Oshukova, Riittakerttu Kaltiala-Heino, Jouko Miettunen, Riikka Marttila, Pekka Tani, Eeva T Aronen, Mauri Marttunen, Matti Kaivosoja, Nina Lindberg

**Affiliations:** Psychiatry, Helsinki University and Helsinki University Hospital, P.O. Box 282, 00029 HUS Helsinki, Finland; University of Tampere, School of Medicine, 33014 Tampere, Finland; Department of Adolescent Psychiatry, Tampere University Hospital, 33380 Pitkäniemi, Finland; Department of Psychiatry, Center for Clinical Neurosciences, University of Oulu and Oulu University Hospital, Oulu, Finland; Medical Research Center Oulu, Oulu University Hospital and University of Oulu, Oulu, Finland; Center for Life Course Epidemiology and Systems Medicine, University of Oulu, Oulu, Finland; Psychiatry, Helsinki University and Helsinki University Hospital, P.O. Box 442, 00029 HUS Helsinki, Finland; Child Psychiatry, Helsinki University and Helsinki University Hospital, P.O. Box 3, 00014 Helsinki, Finland; Adolescent Psychiatry, Helsinki University and Helsinki University Hospital, P.O. Box 3, 00014 Helsinki, Finland; Department of Mental Health and Substance Abuse Services, National Institute for Health and Welfare, P.O. Box 30, 00271 Helsinki, Finland; Department of Child Psychiatry, University of Turku, 20014 Turku, Finland; Hospital District of Central Ostrobothnia, Mariankatu 16-20, 67200 Kokkola, Finland; Forensic Psychiatry, Helsinki University and Helsinki University Hospital, Kellokoski Hospital, 04500 Kellokoski, Finland

**Keywords:** Psychopathic traits, Adolescence, The Youth Psychopathic traits Inventory, The Antisocial Process Screening Device, Psychometric properties

## Abstract

**Background:**

In general psychiatric services, cost-benefit screening instruments for psychopathic traits in adolescents are needed. The aim of the present study was to study the psychometric properties of the Finnish versions of the Youth Psychopathic traits Inventory (YPI) and the Antisocial Process Screening Device (APSD-SR) in community youth. As gender-specific differences exist in psychopathic traits, we analyzed the data separately in girls and boys.

**Methods:**

The YPI and the APSD-SR were administered to 372 9th graders (174 boys and 198 girls) with a mean age of 15.06 years (SD 0.28). Cronbach’s alphas were used to study internal consistency. The factor structures of the self-assessments were studied using both Confirmatory Factor Analysis (CFA) and Principal Component Analysis (PCA).

**Results:**

In both self-assessments, boys scored significantly higher in the total scores, Interpersonal and Affective dimension scores as well as in most sub-dimensions. In the YPI, the alpha values for total and dimensional scores ranged from 0.55 to 0.91 in boys and from 0.74 to 0.89 in girls and, in the APSD-SR, respectively, from 0.38 to 0.78 and from 0.29 to 0.78. In CFA, the three-factor model produced poor fit for both self-assessments. For the ten sub-dimensions of the YPI, the PCA suggested two factors. Extending the model into three components showed sub-dimension loadings according to the original dimensions. For the APSD-SR, the PCA revealed a five-factor structure in the male sample and a six-factor one in the female group. When limiting the model to a three factor- model, we obtained a structure, which resembled the original dimensions.

**Conclusions:**

Both the YPI and the APSD-SR are promising tools of screening for psychopathic features in Finnish community youth. The YPI turned out to be slightly better than the APSD- SR in both reliability and factor structure. However, the original three-factor models did not find support. Both self-assessments were somewhat weak for tapping the callous-unemotional traits of the psychopathic character, but, again, the YPI worked better than the ASPD-SR. Both self-assessments revealed significant gender differences in psychopathic character traits.

**Electronic supplementary material:**

The online version of this article (doi:10.1186/s13034-015-0047-6) contains supplementary material, which is available to authorized users.

## Background

A personality trait is a more or less stable way of experiencing and perceiving oneself and one’s surroundings, as well as relating to others. Deficient interpersonal (superficial charm, grandiose sense of self-worth and manipulation), affective (shallow affect, lack of empathy, lack of remorse or guilt), and behavioral (impulsivity, failure to carry responsibility for one’s own actions) characteristics comprise psychopathic character traits. According to the current conception, psychopathic traits are a continuum of features that each individual exhibits to a certain extent, and psychopathy is a malicious conceptualization of the extremes of normal personality traits [1, 2].

Psychopathic traits are described as relatively stable over time from childhood through adolescence to adulthood [3]. Adolescents with psychopathic traits are stimulus seeking [4], more reactive to reward than punishment [5] and more likely to violate social norms of society and engage in antisocial behavior [6–8]. Besides more severe aggression, youth with elevated psychopathic traits display more instrumental and premeditated aggression compared to other adolescents with severe conduct problems [3]. Furthermore, psychopathic traits are associated with an earlier onset to severe conduct problems [9]. Psychopathic traits in adolescents are strongly related to various mental disorders [10] and drug use [11]. Poor treatment compliance and a high drop-out rate in mental health services have also been linked to high traits of psychopathy [12]. However, recent research has suggested that adolescents with elevated psychopathic traits are not “untreatable” and that they can improve with intensive interventions tailored to their unique emotional, cognitive, and motivational styles [13]. The real challenge for the mental health services is that they should be able to detect these adolescents.

Research on the relative prevalence rates of psychopathic traits in boys and girls is mixed, with some studies reporting overall higher psychopathic tendencies among boys than among girls, and others finding no gender differences [14]. It has been stated, that higher psychopathy scores for boys than for girls tend to emerge in samples recruited from community settings, while studies among justice-involved youth have reported fewer differences in psychopathic scores across the genders [14]. All in all, more research is needed on gender differences in psychopathic character traits in adolescence.

The gold standard for assessing adolescent psychopathic traits is the Psychopathy Checklist- Revised: Youth version [15]. This is, however, a time-consuming method that demands rigorous training and is mainly used in forensic samples. General psychiatric services have a need for cost-beneficial screening instruments for adolescent populations. This need became even more urgent in 2013, as a subtype of conduct disorder characterized by callous-unemotional traits was introduced in the fifth version of the Diagnostic and Statistical Manual of Mental Disorders (DSM 5) [16]. Both the Youth Psychopathic traits Inventory (YPI) [11] and the Antisocial Process Screening Device - Self Report (APSD-SR) [17] are questionnaires designed to assess psychopathic traits among 13- to 18-year-old community youth. Both of these self-assessments measure the interpersonal, affective and behavioral dimensions of psychopathy and have shown acceptable psychometric properties [11, 18–20]. There are, however, some differences between these two self-questionnaires. The APSD-SR contains one item for each of the 20 items measured by the Hare Psychopathy Checklist - Revised [21], the precursor of self-assessments measuring psychopathy, whereas the YPI assesses each psychopathic trait with several items. The APSD-SR tends to ask about psychopathy-like behavior directly (e.g. “I lie easily and skillfully”, “I blame others for my mistakes”), but in the YPI, the items are composed to tap psychopathic traits more indirectly, framing the psychopathic features as abilities, rather than deficits (e.g. “I usually feel calm when other people are scared” instead of “My emotions are shallow”) [11].

The current language versions of the self-assessments are extremely important to study before extensive use since the translation might not capture the meaning of the item adequately and cultural characteristics affect the comprehensiveness of the items. The aim of the present study was to study the psychometric properties of the Finnish versions of the YPI and the APSD-SR in Finnish community youth. As gender-specific differences have been reported in studies performed with these instruments [4, 11, 20, 22] we analyzed the data separately in girls and boys. We hypothesized that, in line with previous studies among justice-involved youth [23, 24], the YPI would appear to be slightly better in tapping psychopathic traits than the APSD-SR. Our second hypothesis was that both self-assessments would reveal significant gender differences.

## Method

### Participants

The sample comprised 15- to 16-year-old Finnish-speaking adolescents attending the 9th grade at secondary schools in the city of Kokkola, on the western coast of Finland, in January 2014. Kokkola is the 23rd largest town in Finland with approximately 47 000 citizens. Eighty-four percent of its citizens speak Finnish, 13 % Swedish and 3 % some other language as their mother tongue.

Of the 446 students in five secondary schools, 60 (13.4 %) did not participate in the study because of either not attending school on the study day or refusing participation. Of the remaining 386 students, eight did not complete the self-assessments and six did not provide the collected background variables asked for in the questionnaire, and thus were excluded from the analysis. The final sample comprised 372 adolescents with a mean age of 15.06 years (SD 0.28), of whom 174 (46.8 %) were boys and 198 (53.2 %) girls.

### Self-assessments

#### The YPI

The YPI [11] consists of 50 statements scored on a 4-point Likert scale with response options ranging from “Does not apply at all = 1” to “Applies very well = 4”; thus, the total score of the scale can range from 50 to 200, with a higher score representing a higher level of the trait. The YPI has three dimensions (factors) and 10 sub-dimensions. The Interpersonal (Grandiose-manipulative) dimension consists of sub-dimensions named Dishonest charm, Grandiosity, Lying and Manipulation, the Affective (Callous-unemotional) dimension of Remorselessness, Unemotionality and Callousness, and the Behavioral (Impulsive-irresponsible) dimension of Thrill-seeking, Impulsiveness and Irresponsibility. All sub-dimensions are scored with five items. In this study we used the authorized Finnish translation of the YPI, which was commissioned by the authors. An iterative process of translation and independent back translation was used, followed by a discussion to resolve minor differences.

#### The APSD-SR

The APSD [17] was originally developed to study children aged 6 to 13 years, but, later, it has been used as a self-assessment (APSD-SR) tool for adolescent populations [23–25]. It consists of 20 statements scored on a 3-point scale (0 = not at all true, 1 = sometimes true, 2 = definitely true), the total score of the scale ranging from 0 to 40, with a higher score representing a higher level of the trait. The three dimensions (factors) of the scale are Interpersonal (Narcissism), Affective (Callous/unemotional) and Behavioral (Impulsivity). In the present study, the authorized Finnish translation of the APSD-SR was used [25].

### Procedure

The present study is a part of an on-going study project investigating psychopathic traits among Finnish adolescents. The adolescents completed the above mentioned self-assessments together with the Youth Self Report (YSR) during their regular school classes. Prior to completing the assessments, they received information about the study both orally and in a cover letter. The participants were assured of the confidentiality and anonymity of the data and of the voluntary nature of participation. Return of the completed questionnaires from the participants was taken as confirmation of their consent. Privacy was ensured by including no identifying factors in the questionnaires; only age and gender were collected as background variables. A letter was sent to the guardians of the students to inform them about the study, and they had the opportunity to familiarize themselves with the self-assessments. The adolescents and parents were informed that the study aimed to investigate adolescents’ thoughts, ideas and feelings towards different aspects of life as well as adolescents’ behavior and well-being. Further, the adolescents were informed that they had an opportunity to contact the researchers (e-mails and telephone numbers were offered) if the content of the self-assessments raised questions or ideas, which they wanted to share with the researchers. The study plan was evaluated by the Ethics Committee of the Helsinki and Uusimaa Hospital District. Permission to conduct the study was granted by the administration of the schools.

### Statistical analyses

In order to evaluate the internal consistency of both the YPI and the APSD-SR, we calculated Cronbach’s alphas for the total and dimensional scores, as well as for the sub-dimensional scores of the YPI. In line with previous research, reliability coefficients of < 0.60 were interpreted as insufficient, 0.60 to 0.69 as marginal, 0.70 to 0.79 as acceptable, 0.80 to 0.89 as good, and 0.90 as excellent [26].

We provided descriptive information concerning the distribution of the YPI and the APSD-SR scores separately for boys and girls. Average continuous scores were reported. According to the skewness and kurtosis, some of the variables were not normally distributed. While looking closer on the distribution skewness, we found that it was mostly due to a small group of participants (three boys and two girls) with very high scores. We also checked the main statistical parameters with omission of these five adolescents, and the omission did not influence the results. So, we proceeded with the whole sample using non-parametric tests. The Mann–Whitney *U*-test was used to test the group differences. We also calculated Cohen’s d to estimate the effect sizes of the gender differences, interpreting an effect size of 0.2 to 0.5 as small, 0.5 to 0.8 as medium, and over 0.8 as large [27]. The convergent validity of the YPI and the APSD-SR was explored by calculating Spearman’s correlations. As recommended [27], we considered a Spearman’s coefficient of 0.1 to 0.3 as small, 0.3 to 0.5 as moderate, and >0.5 as high.

We attempted to replicate the three-component structure of the self-assessments with the Confirmatory Factor Analysis (CFA), performed with the Mplus 7 statistical software [28]. To check the fit of the model to our data, we used the Chi-square Test of model fit for the baseline model, the Comparative Fit Index (CFI), and the Root Square Error of Approximation (RMSEA). The CFI values > 0.90 indicated a reasonably good fit, and in RMSEA, values < 0.06 indicated an acceptable model fit. As the fits assessed using the CFA were not adequate, we performed a Principal Component Analysis (PCA) with oblique Promax rotation to explore the factor structure of the self-assessments. The oblique rotation method was used in line with the previous research [20], as we wanted to let the factors correlate with each other.

For both self-assessments, we checked the number of factors using the Kaiser criterion (i.e. eigenvalues >1) and a scree plot. The three-factor structure was analyzed for both assessments for comparability with the previous studies [11, 20, 25, 29]. According to Kline [30], loadings of 0.30 or higher were considered significant. All the statistical analyses except the CFA were performed with IBM SPSS Statistics version 19.

## Results

### Descriptive information

Table 1 shows the means, standard deviations (SD) and medians of the YPI and the APSD-SR dimensional and total scores as well as the YPI sub-dimensional scores, separately in boys and girls. In both self-assessments, boys scored significantly higher in the total scores as well as in Interpersonal and Affective dimension scores than girls did. Focusing on the sub-dimensions of the YPI, boys scored significantly higher than girls in Grandiosity, Lying, Remorselessness, Unemotionality, Callousness and Irresponsibility. According to the Cohen’s d coefficient, differences were most prominent on the Affective dimension of both self-assessments and on two of the corresponding sub-dimensions of the YPI (Callousness and Unemotionality).

**Table 1:** Descriptives and mean group differences in the Youth Psychopathic traits Inventory (YPI) and the Antisocial Process Screening Device-self-report (APSD-SR) scores between 15- to 16-year-old boys (*n* = 174) and girls (*n* = 198) attending the 9th grade at 5 secondary schools in Finland. Comparisons are performed using the Mann–Whitney *U*-test. Effect sizes (Cohen’s d) are reported

	Boys					Girls					Statistics	
	Mean (SD)	Minimum-maximum	Median	Skewness (SE = 0.184)	Kurtosis (SE = 0.366)	Mean (SD)	Minimum-maximum	Median	Skewness (SE = 0.173)	Kurtosis (SE = 0.344)	Mann–Whitney *U* Test	Cohen’s d
*YPI Sub-dimension*												
Dishonest charm	1.80 (0.68)	1.00-4.00	1.80	0.839	0.579	1.73 (0.66)	1.00-4.00	1.60	0.860	0.309	16083.50	0.104
Grandiosity	1.89 (0.68)	1.00-4.00	1.80	1.040	1.103	1.58 (0.61)	1.00-3.60	1.40	1.079	0.529	12092.50*	0.480
Lying	1.80 (0.67)	1.00-4.00	1.80	0.887	0.687	1.65 (0.61)	1.00-4.00	1.50	0.927	0.381	14955.00**	0.234
Manipulation	1.69 (0.69)	1.00-4.00	1.50	1.075	0.838	1.63 (0.62)	1.00-4.00	1.40	1.058	0.973	16690.50	0.091
Remorselessness	1.69 (0.64)	1.00-4.00	1.60	1.256	1.962	1.44 (0.53)	1.00-4.00	1.40	1.810	4.532	12999.50*	0.425
Unemotionality	2.13 (0.56)	1.00-4.00	2.20	0.306	0.442	1.80 (0.53)	1.00-4.00	1.80	1.319	2.733	10740.00*	0.605
Callousness	2.23 (0.50)	1.00-3.40	2.20	−0.292	−0.135	1.67 (0.47)	1.00-4.00	1.60	0.970	2.183	6988.00*	1.154
Thrill-seeking	2.55 (0.72)	1.00-4.00	2.60	0.021	−0.530	2.49 (0.64)	1.00-4.00	2.60	0.006	−0.322	16349.00	0.088
Impulsiveness	2.14 (0.67)	1.00-4.00	2.00	0.347	−0.348	2.22 (0.67)	1.00-4.00	2.20	0.242	−0.447	15909.50	−0.119
Irresponsibility	1.85 (0.70)	1.00-4.00	1.80	0.754	−0.102	1.60 (0.57)	1.00-3.80	1.40	1.114	1.058	13722.00*	0.392
*YPI Dimension*												
Interpersonal	7.18 (2.38)	4.00-16.00	7.00	0.998	1.635	6.59 (2.10)	4.00-15.40	6.40	1.024	1.381	14645.00**	0.263
Affective	6.05 (1.26)	3.00-11.40	6.00	0.768	1.724	4.92 (1.25)	3.00-12.00	4.60	1.667	5.534	8097.50*	0.900
Behavioral	6.54 (1.84)	3.00-12.00	6.35	0.390	−0.287	6.31 (1.60)	3.20-11.40	6.20	0.414	−0.960	16166.00	0.133
*YPI Total*	19.77 (4.94)	11.80-38.80	19.60	0.876	1.546	17.82 (4.19)	10.40-37.40	17.20	1.257	3.4160	13066.00*	0.426
*APSD-SR Dimension*												
Interpersonal	0.44 (0.40)	0.00-2.00	0.29	1.212	1.650	0.33 (0.32)	0.00-2.00	0.29	1.788	4.992	11552.50*	0.304
Affective	0.73 (0.34)	0.00-1.67	0.67	0.391	−0.041	0.55 (0.28)	0.00-1.67	0.50	1.038	2.083	14392.50*	0.578
Behavioral	0.73 (0.44)	0.00-2.00	0.80	0.423	−0.218	0.68 (0.43)	0.00-2.00	0.80	0.566	0.219	6197.50	0.115
*APSD-SR Total*	1.90 (0.82)	0.37-4.33	1.77	0.526	0.022	1.56 (0.77)	0.31-5.18	1.41	1.284	2.901	12658.00*	0.427

*difference is significant at the 0.01 level (2-tailed); **difference is significant at the 0.05 level (2-tailed)

### Internal consistency

Table 2 presents the Cronbach’s alpha values for the YPI and the APSD-SR total and dimensional scores as well as for the YPI sub-dimensional scores, separately in boys and girls. For the YPI, Cronbach’s alpha coefficient indicated good to excellent internal consistency in boys and girls for the total as well as for both the Interpersonal and Behavioral dimension scores. The Affective dimension score of the YPI showed acceptable internal consistency in girls, but insufficient in boys. Internal consistency was mostly good or at least acceptable for all but three sub-dimensions: Irresponsibility in girls and Unemotionality in both genders showed marginal internal consistency, and Cronbach’s alphas for Callousness in both genders indicated insufficient internal consistency.

**Table 2:** Internal consistencies for the sub-dimensions, dimensions and the total score of the Youth Psychopathic traits Inventory (YPI) and the dimensions and total score of the Antisocial Process Screening Device - self-report (APSD-SR) in 15- to 16-year-old boys (*n* = 174) and girls (*n* = 198) attending the 9^th^ grade in 5 secondary schools in Finland

	Boys	Girls
YPI Dishonest charm	0.81	0.82
YPI Grandiosity	0.78	0.82
YPI Lying	0.82	0.77
YPI Manipulation	0.86	0.80
YPI Remorselessness	0.83	0.79
YPI Unemotionality	**0.65**	**0.65**
YPI Callousness	**0.41**	**0.43**
YPI Thrill-seeking	0.80	0.78
YPI Impulsiveness	0.73	0.77
YPI Irresponsibility	0.74	**0.68**
YPI Interpersonal dimension	0.90	0.86
YPI Affective dimension	**0.55**	0.74
YPI Behavioral dimension	0.86	0.80
YPI Total score	0.91	0.89
APSD-SR Interpersonal dimension	0.78	0.74
APSD-SR Affective dimension	**0.38**	**0.29**
APSD-SR Behavioral dimension	**0.68**	**0.68**
APSD-SR Total score	0.78	0.79

Cronbach’s alpha coefficients are reported. Indices below the recommended value for at least acceptable reliability are in boldface

For the APSD-SR, Cronbach’s alphas showed acceptable internal consistencies for the total score in both genders. As for the dimensional scores, the internal consistency was acceptable for the Interpersonal, marginal for the Behavioral and insufficient for the Affective dimension in both genders.

To further elucidate the reliability of the YPI and the APSD-SR, we examined inter-dimensional and dimension-total score correlations (Table 3). In the YPI, the inter-dimensional correlations and correlations between the total score and each dimension’s score were high in both genders, except the one between the Affective and Behavioral dimension scores in girls, which was only moderate. In the APSD-SR, the correlations between the total and Behavioral dimension scores and between the total and Interpersonal scores were high in both genders. The correlation between the total and Affective dimension scores was moderate in both genders. Further, correlation between the Interpersonal and Behavioral dimension scores was high in boys, but moderate in girls. The correlation between the Interpersonal and Affective dimension scores as well as between the Affective and Behavioral dimension scores was low in girls and negligible in boys.

**Table 3:** Spearman’s correlations between the dimensional and total scores of the Youth Psychopathic traits Inventory (YPI) and the Antisocial Process Screening Device -self report (APSD-SR) in 15-to 16-year-old boys (*n* = 174) and girls (*n* = 198) attending the 9th grade in 5 secondary schools in Finland

	YPI				APSD-SR			
	Interpersonal	Affective	Behavioral	Total	Interpersonal	Affective	Behavioral	Total
	Boys/Girls	Boys/Girls	Boys/Girls	Boys/Girls	Boys/Girls	Boys/Girls	Boys/Girls	Boys/Girls
YPI Dimension								
Interpersonal	-	0.68^**^/0.54^**^	0.69^**^/0.52^**^	0.91^**^/0.88^**^	0.72^**^/0.72^**^	0.06/0.21^**^	0.57^**^/0.47^**^	0.65^**^/0.61^**^
Affective	0.68^**^/0.54^**^	-	0.62^**^/0.38^**^	0.83^**^/0.71^**^	0.56^**^/0.55^**^	0.28^**^/0.47^**^	0.53^**^/0.35^**^	0.64^**^/0.52^**^
Behavioral	0.69^**^/0.52^**^	0.62^**^/0.38^**^	-	0.88^**^/0.79^**^	0.54^**^/0.45^**^	0.05/0.04	0.72^**^/0.77^**^	0.69^**^/0.69^**^
YPI total	0.91^**^/0.88^**^	0.83^**^/0.71^**^	0.88^**^/0.79^**^	-	0.70^**^/0.72^**^	0.08/0.26^**^	0.69^**^/0.65^**^	0.74^**^/0.75^**^
APSD-SR Dimension								
Interpersonal	0.72^**^/0.72^**^	0.56^**^/0.55^**^	0.54^**^/0.45^**^	0.70^*^/0.72^**^	-	0.08/0.25^**^	0.57^**^/0.41^**^	0.74^**^/0.61^**^
Affective	0.06/0.21^**^	0.28^**^/0.47^**^	−0.05/0.04	0.08/0.26^**^	0.08/0.25^**^	-	−0.04/0.18^*^	0.24^**^/0.41^**^
Behavioral	0.57^**^/0.47^**^	0.53^**^/0.42^**^	0.72^**^/0.77^**^	0.69^**^/0.65^**^	0.57^**^/0.41^**^	−0.04/0.18^*^	-	0.80^**^/0.70^**^
APSD-SR total	0.65^**^/0.61^**^	0.64^**^/0.52^**^	0.69^**^/0.69^**^	0.74^**^/0.75^**^	0.74^**^/0.61^**^	0.24^**^/0.42^**^	0.80^**^/0.70^**^	-

*correlation is significant at the 0.05 level (2-tailed); **correlation is significant at the 0.01 level (2-tailed)

### Convergent validity

In Table 3, the correlations between the YPI and the APSD-SR are presented. In both genders, the total scores as well as the Interpersonal and Behavioral dimension scores of the YPI and the APSD-SR were highly correlated with each other, but the correlation between the Affective dimension scores of the two instruments was only weak.

### Factor analysis

The CFA, which was performed for the 10 sub-dimensions of the YPI, did not support the hypothesized factor solution in boys and did not converge in girls. We attempted to replicate the three-factor model on the item level. For both self-assessments, the three-factor model produced a poor fit (YPI: boys/girls: *x*^2^ = 2461/4924, df = 1172/1225, CFI = 0.680/0.670, RMSEA = 0.080/0.072; APSD-SR: boys/girls: *x*^2^ = 610/512, df = 169/169, CFI = 0.475/0.546, RMSEA = 0.123/0.101). The items loaded mostly in a theoretically meaningful way, with the exception of some YPI items constituting the Callousness sub-scale (boys: items 23, 35 and 49; girls: items 35 and 49) and, respectively, some APSD-SR Items constituting the Affective dimension (boys: items 3, 7, 12, 18, and 20; girls: items 3 and 19). In these cases, loading indices were insufficient. For details, see the Additional file 1.

Table 4 shows the results of the exploratory PCA with oblique Promax rotation for the 10 sub-dimensions of the YPI and 20 items of the APSD-SR, both forced into a three component model. For the ten sub-dimensions of the YPI, the PCA suggested two factors with eigenvalues greater than one, covering 67 % (boys) and 65 % (girls) of the total variance. For boys, the two-factor structure was not theoretically interpretable, as all sub-dimensions except Callousness loaded on the same factor. Extending the model into three components showed sub-dimension loadings according to the original dimensions, except that, in boys, Remorselessness and Unemotionality loaded on the interpersonal factor instead of the affective one, and in girls, these same sub-dimensions loaded on both interpersonal and affective factors.

**Table 4:** Loading of the sub-dimensions of the Youth Psychopathic traits Inventory (YPI) and items of the Antisocial Process Screening Device -self report (APSD-SR) into three factors in boys (*n* = 174) and girls (*n* = 198)

YPI Sub-dimension/APSD-SR Item	Interpersonal		Affective		Behavioral	
	Boys	Girls	Boys	Girls	Boys	Girls
YPI						
Dishonest charm	**0.811**	**0.897**	−0.066	−0.107	0.153	0.101
Grandiosity	**0.906**	**0.918**	−0.075	0.136	−0.165	−0.296
Lying	**0.780**	**0.657**	0.008	−0.079	0.089	0.240
Manipulation	**0.935**	**0.887**	−0.005	−0.033	−0.026	0.084
Remorselessness	**0.680**	**0.316**	0.111	**0.480**	0.204	0.276
Unemotionality	**0.640**	**0.335**	0.059	**0.602**	0.006	−0.050
Callousness	−0.012	−0.162	**0.996**	**0.948**	−0.024	−0.007
Thrill-seeking	−0.131	0.105	−0.075	−0.042	**0.988**	**0.902**
Impulsiveness	−0.163	0.069	−0.049	−0.138	**0.782**	**0.872**
Irresponsibility	0.051	−0.202	0.106	0.190	**0.806**	**0.861**
*Eigenvalue*	*5.72*	*5.13*	*1.01*	*1.34*	*0.80*	*0.88*
*Variance explained*	*57 %*	*51 %*	*10 %*	*13 %*	*8 %*	*9 %*
APSD-SR						
5 Shallow emotions	0.225	**0.549**	0.176	0.093	**0.461**	−0.15
8 Brags about accomplishments	**0.748**	**0.693**	−0.213	−0.123	−0.122	−0.181
10 Uses or cons others	**0.536**	**0.589**	0.070	0.064	0.106	0.214
11 Teases other people	**0.613**	**0.499**	0.157	0.151	−0.005	0.037
14 Charming in insincere ways	**0.573**	**0.656**	−0.046	−0.225	0.236	0.148
15 Becomes angry when corrected	**0.420**	**0.470**	−0.023	0.021	**0.380**	0.201
16 Thinks he is more important	**0.887**	**0.801**	0.012	−0.019	−0.263	−0.188
1 Blames others for mistakes	**0.600**	0.145	0.177	0.256	−0.018	**0.312**
9 Gets bored easily	−0.044	0.118	−0.226	−0.234	**0.550**	**0.521**
4 Acts without thinking	0.021	−0.021	0.048	0.046	**0.696**	**0.743**
13 Engages in risky and dangerous behavior	−0.144	0.115	−0.039	−0.085	**0.868**	**0.694**
17 Does not plan ahead	−0.053	0.005	−0.108	−0.018	**0.661**	**0.507**
19 Does not show emotions	**0.514**	−0.061	−0.186	0.033	0.013	**0.578**
7 Keeps promises (Reversely = R)	0.026	−0.003	**0.641**	**0.661**	−0.151	0.053
12 Feels bad or guilty	0.021	0.294	**0.725**	**0.387**	0.057	−0.127
18 Concerned about the feelings of others (R)	−0.046	0.132	**0.722**	**0.722**	−0.005	−0.142
20 Keeps the same friends (R)	−0.027	−0.309	**0.674**	**0.760**	−0.151	0.013
3 Concerned about schoolwork(R)	0.061	0.282	−0.036	0.044	**−0.659**	**−0.813**
6 Lies easily and skillfully	**0.408**	**0.318**	−0.024	0.238	**0.481**	**0.340**
2 Engages in illegal activities	−0.077	0.189	0.126	0.81	**0.864**	**0.595**
*Eigenvalue*	*5.71*	*5.14*	*2.17*	*1.83*	*1.68*	*1.56*
*Variance explained*	*29 %*	*26 %*	*11 %*	*9 %*	*8 %*	*8 %*

Principal Component Analysis with oblique Promax rotation was used. Significant loading indices are in bold

For the APSD-SR, the PCA revealed a five-factor structure in boys and a six-factor one in girls, with eigenvalues greater than one, accounting for 59 and 60 % of the total variance, respectively. When limiting the model to three-factor model, we obtained a structure which resembled the original dimensions. However, in both genders, item six (Lies easily and skillfully) loaded on two factors (interpersonal and behavioral). Further, item one (Blames others for mistakes) loaded on the interpersonal factor in boys, but on the behavioral one in girls, item five (Shallow emotion) on the behavioral factor in boys, but on the interpersonal one in girls, item 15 (Becomes angry when corrected) on the interpersonal factor in girls, but on both interpersonal and affective ones in boys, and item 19 (Does not show emotions) on the interpersonal factor in boys, but on the behavioral one in girls. Item three (Concerned about schoolwork, coded reversely) loaded negatively on the behavioral factor in both genders.

## Discussion

### Main findings

This study was to first to study the psychometric properties of the Finnish versions of the YPI and the APSD-SR in the same adolescent community sample. Previous research has revealed that the YPI is internally consistent among non-referred boys and girls [11, 20]. Our study largely supported these previous results, since we found good or even excellent internal consistencies of the YPI total as well as Interpersonal and Behavioral dimension scores in both genders. However, in line with some previous studies performed both in community and forensic samples [20, 31, 32], the internal consistency of the Affective dimension was only acceptable in girls and even insufficient in boys. Correlations between the total and dimensional scores proved to be strong.

In a recent community study using the APSD-SR by Pechorro et al. [33] among 510 mid-adolescent Portuguese pupils, the internal consistency was acceptable for the total score, marginal for the interpersonal dimension, but insufficient for the affective and behavioral dimensions. Accordingly, in our study, Cronbach’s alpha showed acceptable internal consistency for the total score and insufficient internal consistency for the Affective dimension in both genders. However, in the present study, the internal consistency was acceptable for the Interpersonal and marginal for the Behavioral dimensions. A recent Finnish community study among 4855 9^th^ graders by Laajasalo et al. [25] reported internal consistency indices for the APSD-SR total as well as for the Interpersonal and Behavioral dimension scores, which were highly consistent with our results (α: 0.70–0.76). However, in their study, the Affective dimension exhibited better reliability (α = 0.67) than was seen in our work. Focusing on correlations, both in our study and in the study by Laajasalo et al., the Affective dimension score correlated only modestly with the two other dimensional scores.

### Comparison between the YPI and the APSD-SR

As far as the authors are aware, only two previous studies have compared the psychometric properties of the YPI and the APSD-SR in adolescents [23, 24]. Both studies were performed among delinquents and reported that both self-assessments were somewhat weak for tapping the affective traits of psychopathy; however, the YPI appeared to be slightly better than the APSD-SR. Our results with mid-adolescent community youth support this finding. It has been argued that the reason for this might be the substantial differences between the scales: the YPI comprises more items and taps psychopathic traits more indirectly than the APSD-SR [23, 24]. Interestingly, in the present study, and in line with that of Colins et al. [24], the YPI Interpersonal dimension was strongly related to the Affective dimension, as well as to the Behavioral one. What comes to APSD-SR dimensions, only the latter correlation was significant. Thus, the question rises whether the APSD-SR rather taps antisocial behavior features than the other elements of psychopathic character traits. This idea is not new, as the need for further revision has been stated concerning the APSD-SR [24]. The developers of the YPI have also proposed that the current number of items, even though higher than in the APSD-SR, is not high enough to detect the sub-dimensions comprising the Affective dimension and, because of this, the instrument may need further revision [32]. In addition, the development of new instruments is an important area of the future work. One unpleasant thought is, however, that the affective dimension of psychopathic character traits may simply be too difficult to self-evaluate among adolescents. If this is the case, the self-assessment should always be strengthened by a clinical interview. All in all, the screening instrument’s ability to assess the affective dimension is extremely important since, according to many researchers, it is the affective and interpersonal features that comprise the “core” of the psychopathy [11, 34], foreshadowing a great risk of long-term maladjustment in children and adolescents [35, 36].

### Gender differences

Some researchers have reported higher psychopathic tendencies among adolescent boys than among girls, both in community and forensic samples, and others have found no gender differences or differences in certain traits. These inconsistent findings may be dependent upon the developmental period of the studied adolescents, the study method, the sample selection and the particular dimension of psychopathy being assessed [14]. In line with a recent community study performed using the YPI among Dutch 9^th^ graders [20], our study revealed significantly higher total as well as Interpersonal and Affective dimension scores in boys than in girls. However, the Dutch study reported that boys scored significantly higher also in the Behavioral dimension, which we were not able to find. According to a recent study by Asgeirsdottir and Sigfusdottir [37], girls in Nordic countries tend to report higher levels of anger symptoms than boys. Whether the observed lack of gender difference in antisocial behavior reflects social, educational and economic gender equality characteristics of all Nordic countries, comprises an interesting question to be studied in the future. In our sample, no gender difference was observed on Manipulation and Dishonest charm, both reflecting the Interpersonal dimension of the YPI. Among the Dutch sample, boys scored significantly higher on these items also [20]. These findings are interesting, since deliberate manipulation of peer relationships by, for example, ostracism, gossiping and telling lies about the victims has been seen as a typical female phenomenon, and among adults, the prototypical psychopathic woman exhibits more manipulation than the prototypical psychopathic man does [38]. All in all, cultural aspects seem to have an impact on gender differences in psychopathic character traits already in adolescence.

In our study, the same gender differences emerged in the APSD-SR total as well as in the dimensional scores observed in the YPI, which speaks for the validity of the two measures. We were not able to find studies focusing on gender differences in APSD-SR scores in mid-adolescent community youth, but in a study performed using the teacher and parent versions of the APSD among children with a mean age of 10.7 years, boys scored higher on all three dimensions [29]. The authors reported that the difference was stronger in older grade cohorts. However, Vitacco et al. [39] found no gender differences in a sample of delinquent youth. Again, Poythress et al. [23] were not able to find any gender differences on either the APSD-SR or the YPI in a sample of justice-involved adolescents with a mean age of 14.4 years. Overall, findings on gender differences have been inconsistent depending upon the developmental stage of the studied individuals, the study method, the sample selection, and the particular dimension of psychopathy being assessed [14]. Studies among adult samples have, however, repeatedly revealed that significant differences between genders exist [38].

### Factor analysis of the YPI and the APSD-SR

When studying the psychometric properties of the inventories, the factor structure analysis is of great importance, since replication of the factor structure in different samples increases confidence in the usefulness of the composite scores [40]. We chose the three-factor model for the CFA, because the current literature on the factor structure mostly discusses three or four factors underlying the psychopathy construct [33, 41, 42], and the three-component structure of both the YPI and the APSD-SR has been demonstrated [11, 17, 20, 25]. For both self-assessments, and in both genders, the three-factor model produced a poor fit. However, the fit indices for the YPI proved to be slightly better than those for the APSD-SR. Further, for both measures, the fit was slightly better in girls than in boys. One explanation of our, to some extent unexpected, results with the poor fit might be the relatively small sample size. Though the YPI three-factor structure has received confirmation in numerous studies using the CFA [11, 22, 24], in a study by Poythress et al. [23] in a sample of justice-involved adolescents, the CFA results indicated only a marginal fit of the three-factor model for the data.

In the YPI, the PCA suggested a structure of two factors with eigenvalues greater than one. This was not, however, theoretically interpretable in boys, among whom all sub-dimensions except Callousness loaded on the same factor*.* The three-component model, even though statistically weaker, showed sub-dimension loadings similar to that recommended by the developers of the YPI [11], although in boys, the sub-dimensions Unemotionality and Remorselessness loaded on the interpersonal factor instead of the affective one. In girls, these two sub-dimensions loaded on both the interpersonal and affective factors, which has been reported previously [11, 20]. In a study by Hillege et al. [20], in boys, Lying loaded on both interpersonal and behavioral factors, but this was not observed in the present sample.

On the APSD-SR, the PCA revealed a five-factor structure in boys and a six-factor in girls. When the model was forced into three factors, we received a loading structure, which resembled that reported in the original study [29]. In a Finnish community study [25], the three-factor model produced a close to an adequate fit, and the exploratory factor analysis confirmed three conceptually meaningful factors, resembling previously found ones. In our study, unlike in studies by Frick et al. [29] and Vitacco et al. [39], item three, reversely coded “Concerned about schoolwork”, did not load on the affective factor as expected, but instead negatively on the behavioral factor. Interestingly, this same finding was observed by Laajasalo et al. [25]. The authors suggested as one possible explanation that the Finnish school system is less test-driven compared with many other countries. Item nineteen, “Does not show emotions”, loaded on the interpersonal factor in boys, but on the behavioral one in girls. According to earlier research, this item has exhibited poor performance characteristics as well as low factor loadings [25, 39], and it has even been excluded from the three-factor model due to poor fit [23]. All in all, multiple inconsistent loadings of the APSD-SR items, observed also in the present study, have raised concern since interpretation of the dimensional scores is difficult if the items, which comprise the dimension do not neatly cluster into it [23, 24, 39]. It is difficult to interpret to what extent our findings reflect actual problems in loading and to what extent gender differences. We were not able to find studies focusing on gender differences in the APSD-SR factor structure in mid-adolescent community youth, but in the work of Frick et al. [29], among children with a mean age of 10.7 years, less clear differentiation between the interpersonal and behavioral items was observed in girls than in boys, with many items showing double loadings. Obviously, more research is needed to compare the factor structure of both self-assessments between the genders.

Obviously, more research is needed to explore the factor structure of both self-assessments in larger samples, across gender and culture.

### Strengths and limitations

An obvious strength of the present study is the good participation rate and the sample distribution, with an almost equal number of girls and boys. However, all respondents were 15–16 years old, and the findings cannot be generalized to other age groups. Further, the adolescents were not clinically interviewed, and the ratings were based on self-reports. Moreover, the sample size was relatively small, which might explain the poor results of the factor analyses. The variance distribution for some items showed asymmetry. To avoid result bias caused by distribution skewness, we used non-parametric tests. Future studies in adolescent samples with different age ranges, cultures and ethnicity are obviously needed. The present study can be seen as a preliminary validation study and future studies among Finnish adolescent psychiatric patients should be performed before the self-questionnaires are put into use in adolescent psychiatric services.

## Conclusions

Both the YPI and the APSD-SR are promising tools to screen for psychopathic features in Finnish community youth. Among non-referred mid-adolescents, the YPI was slightly better than the APSD- SR in both reliability and factor structure, though the original three-factor models did not gain support. Unfortunately, both self-assessments were somewhat weak for tapping the callous-unemotional traits of the psychopathic character; however, the YPI worked better than the ASPD-SR. Both self-assessments revealed significant gender differences in psychopathic character traits.

## Additional file

Additional file 1:
**Loading of the items of the Youth Psychopathic traits Inventory (YPI) and the Antisocial Process screening Device -self report (APSD-SR) into three factors in boys (** ***n*** **= 174) and girls (** ***n*** **= 198).** The results of the Confirmatory Factor Analysis are presented.
