# Supplementary material for: Self-reported psychopathic traits among non-referred Finnish adolescents: psychometric properties of the Youth Psychopathic traits Inventory and the Antisocial Process Screening Device
Source: Child Adolesc Psychiatry Ment Health. 2015 Jun 6;9:15. doi: 10.1186/s13034-015-0047-6 (PMC4465164; doi:10.1186/s13034-015-0047-6)
Supplement: Additional file 1: — Loading of the items of the Youth Psychopathic traits Inventory (YPI) and the Antisocial Process screening Device -self report (APSD-SR) into three factors in boys ( n = 174) and girls ( n = 198). The results of the Confirmatory Factor Analysis are presented. [file 13034_2015_47_MOESM1_ESM.docx]

| **Additional file.** Loading of the items of the Youth Psychopathic traits Inventory (YPI) and the Antisocial Process screening Device -self report (APSD-SR) into three factors in boys (n= 174) and girls (n= 198). The results of the Confirmatory Factor Analysis are presented. | | | | | | |
| --- | --- | --- | --- | --- | --- | --- |
|  | Interpersonal Dimension | | Affective Dimension | | Behavioral DImension | |
|  | Boys | Girls | Boys | Girls | Boys | Girls |
| **YPI** |  |  |  |  |  |  |
| **Dishonest charm** |  |  |  |  |  |  |
| 6. It's easy for me to charm and seduce others to get what I want from them. | **0.644** | **0.540** |  |  |  |  |
| 33. Pretty often I act charming and nice, even with people I don't like, in order to get what I want. | **0.766** | **0.680** |  |  |  |  |
| 14. I have the ability to con people by using my charm and smile. | **0.676** | **0.705** |  |  |  |  |
| 38. When I need to, I use my smile and my charm to use others. | **0.716** | **0.620** |  |  |  |  |
| 27. When someone asks me something, I usually have a quick answer that sounds believable, even if I've just made it up. | **0.614** | **0.628** |  |  |  |  |
| **Grandiosity** |  |  |  |  |  |  |
| 10. I'm better than everyone on almost everything. | **0.502** | **0.584** |  |  |  |  |
| 37. I'm more important and valuable than other people. | **0.575** | **0.537** |  |  |  |  |
| 41. I am destined to become a well-known, important and influential person. | **0.551** | **0.587** |  |  |  |  |
| 19. I have talents that go far beyond other people's. | **0.448** | **0.599** |  |  |  |  |
| 30. The world would be a better place if I were in charge. | **0.490** | **0.484** |  |  |  |  |
| **Lying** |  |  |  |  |  |  |
| 43. Sometimes I find myself lying without any particular reason. | **0.598** | **0.523** |  |  |  |  |
| 24. Sometimes I lie for no reason, other than because it's fun. | **0.679** | **0.537** |  |  |  |  |
| 50. I've often gotten into trouble because I've lied too much. | **0.614** | **0.465** |  |  |  |  |
| 47. I like to spice up and exaggerate when I tell about something. | **0.653** | **0.636** |  |  |  |  |
| 7. It's fun to make up stories and try to get people to believe them. | **0.674** | **0.588** |  |  |  |  |
| **Manipulation** |  |  |  |  |  |  |
| 15. I am good at getting people to believe in me when I make something up. | **0.639** | **0.631** |  |  |  |  |
| 31. To get people to do what I want, I often find it efficient to con them. | **0.732** | **0.641** |  |  |  |  |
| 11. I can make people believe almost anything. | **0.738** | **0.720** |  |  |  |  |
| 46. It has happened that I’ve taken advantage of (used) someone in order to get what I want. | **0.709** | **0.610** |  |  |  |  |
| 20. It’s easy for me to manipulate people. | **0.794** | **0.701** |  |  |  |  |
| **Remorselessness** |  |  |  |  |  |  |
| 44. To feel guilty and remorseful about things you have done that have hurt other people is a sign of weakness. |  |  | **0.717** | **0.655** |  |  |
| 8. I have the ability not to feel guilt and regret about things that I think other people would feel guilty about. |  |  | **0.711** | **0.592** |  |  |
| 28. When someone finds out about something that I’ve done wrong, I feel more angry than guilty. |  |  | **0.633** | **0.615** |  |  |
| 48. To feel guilt and regret when you have done something wrong is a waste of time. |  |  | **0.775** | **0.676** |  |  |
| 21. I seldom regret things I do, even if other people feel that they are wrong. |  |  | **0.679** | **0.646** |  |  |
| **Unemotionality** |  |  |  |  |  |  |
| 2. I usually feel calm when other people are scared. |  |  | 0.239 | **0.342** |  |  |
| 36. What scares others usually doesn’t scare me. |  |  | **0.450** | **0.320** |  |  |
| 25. To be nervous and worried is a sign of weakness. |  |  | **0.578** | **0.644** |  |  |
| 45. I don’t let my feelings affect me as much as other people’s feelings seem to affect them. |  |  | **0.583** | **0.545** |  |  |
| 39. I don’t understand how people can be touched enough to cry by looking at things on TV or movie. |  |  | **0.520** | **0.515** |  |  |
| **Callousness** |  |  |  |  |  |  |
| 12. I think that crying is a sign of weakness, even if no one sees you. |  |  | **0.624** | **0.304** |  |  |
| 17. When other people have problems, it is often their own fault, therefore, one should not help them. |  |  | **0.461** | **0.536** |  |  |
| 35. I often become sad or moved by watching sad things on TV or film.(Reversely = R) |  |  | -0.197 | 0.236 |  |  |
| 49. I usually become sad when I see other people crying or being sad (R) |  |  | -0.142 | 0.265 |  |  |
| 23. It’s important to me not to hurt other people’s feelings.(R) |  |  | -0.089 | **0.372** |  |  |
| **Thrill-seeking** |  |  |  |  |  |  |
| 1. I like to be where exciting things happen. |  |  |  |  | **0.493** | **0.522** |
| 22. I like to do things just for the thrill of it. |  |  |  |  | **0.733** | **0.751** |
| 42. I like to do exciting and dangerous things, even if it is forbidden or illegal. |  |  |  |  | **0.736** | **0.712** |
| 29. I get bored quickly by doing the same thing over and over. |  |  |  |  | **0.553** | **0.580** |
| 4. I get bored quickly when there is too little change. |  |  |  |  | **0.538** | **0.502** |
| **Impulsiveness** |  |  |  |  |  |  |
| 3. I prefer to spend my money right away rather than save it. |  |  |  |  | **0.567** | **0.411** |
| 26. If I get the chance to do something fun, I do it no matter what I had been doing before. |  |  |  |  | **0.595** | **0.619** |
| 32. It often happens that I do things without thinking ahead. |  |  |  |  | **0.723** | **0.723** |
| 18. It often happens that I talk first and think later. |  |  |  |  | **0.542** | **0.628** |
| 9. I consider myself as a pretty impulsive person. |  |  |  |  | **0.541** | **0.589** |
| **Irresponsibility** |  |  |  |  |  |  |
| 5. I have probably skipped school or work more than most other people. |  |  |  |  | **0.534** | 0.234 |
| 40. I often don't/didn’t have my school or work assignments done on time. |  |  |  |  | **0.616** | **0.521** |
| 13. If I won a lot of money in the lottery I would quit school or work and just do things that are fun. |  |  |  |  | **0.576** | **0.564** |
| 16. I have often been late to work or classes in school. |  |  |  |  | **0.612** | **0.400** |
| 34. It has happened several times that I've borrowed something and then lost it. |  |  |  |  | **0.473** | **0.319** |
| A**PSD-SR** |  |  |  |  |  |  |
| 5. Shallow emotions | **0.567** | **0.661** |  |  |  |  |
| 8. Brags about accomplishments | **0.466** | **0.389** |  |  |  |  |
| 10. Uses or cons others | **0.595** | **0.660** |  |  |  |  |
| 11. Teases other people | **0.489** | **0.518** |  |  |  |  |
| 14. Charming in insincere ways | **0.687** | **0.496** |  |  |  |  |
| 15. Becomes angry when corrected | **0.641** | **0.511** |  |  |  |  |
| 16. Thinks he is more important | **0.538** | **0.646** |  |  |  |  |
| 1. Blames others for mistakes |  |  |  |  | **0.436** | **0.488** |
| 9. Gets bored easily |  |  |  |  | **0.443** | **0.426** |
| 4. Acts without thinking |  |  |  |  | **0.661** | **0.704** |
| 13. Engages in risky and dangerous behavior |  |  |  |  | **0.655** | **0.548** |
| 17. Does not plan ahead |  |  |  |  | **0.557** | **0.382** |
| 19. Does not show emotions |  |  | **0.307** | 0.243 |  |  |
| 7. Keeps promises (Reversely= R) |  |  | 0.075 | **0.514** |  |  |
| 12. Feels bad or guilty |  |  | 0.033 | **0.403** |  |  |
| 18. Concerned about the feelings of others (R) |  |  | 0.034 | **0.682** |  |  |
| 20. Keeps the same friends (R) |  |  | -0.069 | **0.464** |  |  |
| 3. Concerned about schoolwork(R) |  |  | -0.382 | -0.190 |  |  |
|  |  |  |  |  |  |  |
